# A Spontaneous Mutation in Contactin 1 in the Mouse

**DOI:** 10.1371/journal.pone.0029538

**Published:** 2011-12-29

**Authors:** Muriel T. Davisson, Roderick T. Bronson, Abigail L. D. Tadenev, William W. Motley, Arjun Krishnaswamy, Kevin L. Seburn, Robert W. Burgess

**Affiliations:** 1 The Jackson Laboratory, Bar Harbor, Maine, United States of America; 2 Department of Molecular and Cellular Biology, Harvard University, Cambridge, Massachusetts, United States of America; National Institutes of Health, United States of America

## Abstract

Mutations in the gene encoding the immunoglobulin-superfamily member cell adhesion molecule contactin1 (*CNTN1*) cause lethal congenital myopathy in human patients and neurodevelopmental phenotypes in knockout mice. Whether the mutant mice provide an accurate model of the human disease is unclear; resolving this will require additional functional tests of the neuromuscular system and examination of *Cntn1* mutations on different genetic backgrounds that may influence the phenotype. Toward these ends, we have analyzed a new, spontaneous mutation in the mouse *Cntn1* gene that arose in a BALB/c genetic background. The overt phenotype is very similar to the knockout of *Cntn1*, with affected animals having reduced body weight, a failure to thrive, locomotor abnormalities, and a lifespan of 2–3 weeks. Mice homozygous for the new allele have CNTN1 protein undetectable by western blotting, suggesting that it is a null or very severe hypomorph. In an analysis of neuromuscular function, neuromuscular junctions had normal morphology, consistent with previous studies in knockout mice, and the muscles were able to generate appropriate force when normalized for their reduced size in late stage animals. Therefore, the *Cntn1* mutant mice do not show evidence for a myopathy, but instead the phenotype is likely to be caused by dysfunction in the nervous system. Given the similarity of CNTN1 to other Ig-superfamily proteins such as DSCAMs, we also characterized the expression and localization of *Cntn1* in the retinas of mutant mice for developmental defects. Despite widespread expression, no anomalies in retinal anatomy were detected histologically or using a battery of cell-type specific antibodies. We therefore conclude that the phenotype of the *Cntn1* mice arises from dysfunction in the brain, spinal cord or peripheral nervous system, and is similar in either a BALB/c or B6;129;Black Swiss background, raising a possible discordance between the mouse and human phenotypes resulting from *Cntn1* mutations.

## Introduction

This paper reports a spontaneous mutation in the mouse contactin 1 (*Cntn1*) gene (MGI: 105980). CNTN1 is an immunoglobulin (Ig) superfamily member cell adhesion molecule of 1020 amino acids, consisting of six Ig domains, four fibronectin-like repeats (FN), and a GPI-linkage at the carboxy-terminus anchoring the protein to the extracellular plasma membrane [1]. The gene is expressed broadly and robustly in the central and peripheral nervous system. Like many Ig-superfamily proteins, CNTN1 is implicated in numerous neurodevelopmental functions *in vitro*, including neurite extension, repulsion, and fasciculation [2]–[7]. Furthermore, CNTN1 interacts with several extracellular proteins, including other Ig-domain proteins, tenascins, phosphacan, and contactin-associated proteins (Casprs) [4], [8]–[14].

In humans, mutations in *CNTN1* cause a familial form of lethal congenital myopathy (Compton-North congenital myopathy, OMIM ID# 612540), a disease characterized by congenital onset muscle weakness and myopathic features in biopsy samples [15]. There is also a secondary loss of syntrophin and dystrobrevin immunoreactivity from neuromuscular junctions (NMJs) of affected individuals [16]. This condition was mapped to Chromosome 12, and a frame shift mutation in *CNTN1* introducing a premature stop codon (S291fsX296) was identified by sequencing candidate genes within the genetic interval. Consistent with the disease presentation, CNTN1 is found at NMJs in both humans and mice [15].

In mice, the *Cntn1* gene has been deleted by targeting exon 3 using homologous recombination, and the mutation was maintained in a mixed 129/SvJ×C57BL/6×Black Swiss genetic background [17]. Mice lacking *Cntn1* do not have obvious myopathy or defects in syntrophin and dystrobrevin localization at NMJs, and NMJ morphology is normal in the mutant animals [15]. However, *Cntn1* clearly does serve an important neurodevelopmental function in mice. Mice lacking *Cntn1* have ataxia, fail to thrive, and die within 2–3 weeks of birth. Defects in both axon and dendrite development in the cerebellum were observed [17]. In addition, in the peripheral nervous system, CNTN1 is found at the paranodal axolemma, and mice lacking *Cntn1* have reduced nerve conduction velocities and mislocalization of Kv1.1 and Kv1.2 potassium channels [18]. Given the gene's widespread neuronal expression and severity of the *Cntn1* knockout phenotype, it seems likely that there are additional neurological phenotypes in the *Cntn1* mice.

The studies in mice have confirmed that mutations in *Cntn1* cause a severe, early onset phenotype. However, it is unclear whether these mice are a valid model of congenital myopathy. It is possible that there are functional deficits in muscle or neuromuscular junctions that are not evident by histology and immunocytochemistry. It is also possible that different *Cntn1* alleles or different genetic backgrounds could change the phenotype so that it more closely resembles the human disease.

We have begun to address these issues by studying a new, spontaneous mutation in *Cntn1* that arose on an inbred BALB/c genetic background. In these mice we examined NMJs and muscle function to assess this new mutation as a model for congenital myopathy. Furthermore, based on the similarity of CNTN1 to other Ig-superfamily members such as the DSCAMs, we have examined the retina for developmental phenotypes.

## Materials and Methods

### Mice

The *Cntn1* mutant was discovered in The Jackson Laboratory Production colony of BALB/cJ in 1979 by animal care technician Lynn O'Neal. To improve reproduction the mutation was outcrossed once to mice of the BALB/cByJ genetic background. All mice were maintained and crosses carried out in the Mouse Mutant Resource (MMR) at The Jackson Laboratory [19]. Mice are maintained in a room with HEPA-filtered air and a 14∶10 light∶dark cycle. They currently are fed 5K52 6% fat diet (LabDiet.com) and acidified (pH 2.5–3.0) water *ad libitum*. All studies were performed in accordance with the National Institutes of Health Guide for the Care and Use of Laboratory Animals (National Resource Council), and all procedures were approved by The Jackson Laboratory Institutional Animal Care and Use Committee. Comprehensive protocol #01026 (approval dates Nov. 29, 2007 and Oct. 28, 2010) covered the portion of the study done in Dr. Burgess' laboratory and comprehensive protocol #99066 (approval dates July 8, 2010 and June 21, 2011) covered the portion of the study done in Dr. Davisson's laboratory.

### Genetic analysis

Genetic mapping of this new mutation spans the history of mutant gene mapping in the mouse. In the early 1980s, the mutation was tested for chromosomal linkage with visible and polymorphic isoenzyme markers on 15 different chromosomes using intercrosses with linkage testing stocks (stocks carrying multiple mutations that cause visible phenotypes). In linkage crosses and tests for allelism, all mice were classified visually between 10 and 20 days of age. Intercross linkage estimations were calculated using a computer program based on Fisher's Tables [20]. In 1999 the mutant was recovered from the cryopreserved state for more studies and in 2009–2010 mapped more precisely using an intercross with DBA/2J and DNA markers – simple sequence and single nucleotide polymorphisms (SSLPs, SNPs). A female transplanted with homozygous ovaries was mated to a DBA/2J male and obligate heterozygous F1s intercrossed. In this intercross, homozygous mutants were genotyped and the data analyzed as backcross data, giving two chromosomes per mutant mouse.

### Histological analysis

Mice were euthanized with CO_2_ asphyxiation. Tissues from mutant and control littermate mice were fixed in Bouin's fixative, paraffin embedded, sectioned at 4 µm, and stained with hematoxylin and eosin (H&E) in an automated slide processor (Leica). Frozen sections of livers were stained with oil red O to determine if the pale appearance resulted from fat deposition.

### Immunostaining

Cerebellar Purkinje cells were stained with an antibody against calbindin (rabbit anti-calbindin, Chemicon, D-28K at 1∶400) using tissue prepared as above for histology. Signal detection used an avidin-biotin-amplified peroxidase-coupled secondary antibody and Diaminobenzidine chromogenic substrate (Vector Labs) with hematoxylin counterstaining of nuclei.

Retinas were stained using indirect immunofluorescence. Tissue was fixed in 4% buffered paraformaldehyde, cryoprotected in 30% sucrose, and embedded in OCT medium. Samples were then sectioned on a cryostat and mounted onto glass slides for staining. The following primary antibodies were used: Smi-32 mouse anti-non-phosphorylated neurofilament (Covance, SMI-32R at 1∶500), Rabbit anti-melanopsin (generously provided by Ignacio Provencio, Uniformed Services University of the Health Sciences, at 1∶10,000), sheep anti-Tyrosine hydroxylase at 1∶1000 (Millipore AB1542), goat anti-ChAT (Millipore, AB144P at 1∶500), rabbit anti-PKCa (Sigma-Aldrich, P4334 at 1∶1000), rabbit anti-bNOS (Sigma-Aldrich, 1∶1000), rabbit anti-disabled (generously provided by Dr. Brian Howell, at 1∶500), and goat anti-CNTN1 (R&D Systems, AF904). Appropriate secondary antibodies conjugated to AlexaFluor dyes for visualization were used (Invitrogen) with the exception of anti-sheep secondaries, which were Cy3 conjugated (Jackson Immunoresearch). Images were collected on a Zeiss Axio Observer Z1 inverted microscope with a Zeiss Axio Cam digital camera using the software's extended focus function.

Muscle cross sections were prepared and stained as above using rabbit polyclonal antibodies against dystrobrevin and syntrophin (generously provided by Dr. Marvin Adams, University of Washington). The following antibodies were used, anti-α1-syntrophin (258 at 1∶200), anti-β1-syntrophin (248 at 1∶100), anti-β2-syntrophin (149 at 1∶50), anti-α-dystrobrevin (pan alpha, recognizing α-dystrobrevin 1 and 2, 141 at 1∶1000), and anti-β-dystrobrevin (143 at 1∶1000). AlexaFluor 488 conjugated anti-rabbit secondary antibodies were used for visualization. Neuromuscular junctions were counterstained with AlexaFluor 594 conjugated α-bungarotoxin to label nicotinic acetyl choline receptors at the synapse (Invitrogen).

Neuromuscular junctions were visualized as described [21]. In brief, muscles were dissected and fixed in freshly made 4% buffered paraformaldehyde at 4°C for 4 hours. After rinsing in PBS, tissue was partially teased to loosen the muscle fibers, blocked, and permeabilized in PBS with 1% triton ×100 and 2% Bovine Serum Albumin for 30 minutes. During this step, muscles were compressed between two microscope slides with pressure from a binder clip to flatten the muscle to improve imaging. A cocktail of anti-neurofilament (2H3) and anti-SV2 (both from Developmental Studies Hybridoma Bank) were applied in PBS/triton/BSA overnight. Samples were washed extensively the next day and an anti-mouse IgG1 specific secondary antibody conjugated to AlexaFluor 488 was used to visualize nerves. Acetylcholine receptors on the muscle were visualized with α-bungarotoxin conjugated to AlexaFluor 594 (Invitrogen), which was included with the secondary antibody. After twelve hours, samples were washed and mounted in 80% glycerol and imaged on a Leica SP5 confocal microscope.

### *Cntn1* expression in the retina

The expression of *Cntn1* in the mouse retina was examined by *in situ* hybridization. Digoxygenin-labeled (DIG) antisense riboprobes recognizing *Cntn1* were transcribed using T7 polymerase from full length *Cntn1* cDNA. Probes were then subjected to alkaline hydrolysis at pH8.4 at 60°C for 10 minutes to produce probe fragments (∼200 bp), precipitated, resuspended in hybridization buffer and stored at −80°C until use. Retinal tissue sections cut at 20 µm were postfixed in methanol (20 minutes, −20°C), treated with proteinase K (10 minutes, RT), permeabilized with 1% triton-X (30 minutes, RT), and then treated with 0.3% H_2_O_2_ to block endogenous peroxidases. Next, tissue sections were immersed in hybridization buffer without probe for 1 hr at RT and then immersed in hybridization buffer with probe overnight at 65°C. The following day, sections were washed in sodium citrate buffer for 3–4 hrs at 65°C, rinsed in 0.3%TX in PBS and processed for visualization. Signals were detected using an alkaline phosphate-conjugated anti-DIG antibody and NBT colormetric detection, or horseradish peroxidase-conjugated anti-DIG and tyramide signal amplification for fluorescent double label *in situ*s, both according to the manufacturers protocols (Roche).

Immunofluorescence of anti-CNTN1 was performed using a goat-anti-CNTN1 antibody (R&D Systems, AF904, 1∶100). To optimize antigenicity and reduce non-specific labeling, several fixatives were tried, including paraformaldehyde prior to cryo-embedding and sectioning, and methanol, ethanol/acetic acid, or methanol/acetone fixation on cryostat-cut sections of fresh-frozen tissue. Results varied with the fixative, but were generally comparable. Acetic acid/ethanol fixation is shown based on the strong signals in control tissue and the complete absence of signals with the omission of the primary antibody (not shown). All mutant and control samples were processed in parallel and all images were collected under equivalent confocal parameters or camera exposure times.

### Candidate gene analysis

Based on the chromosomal location on Chromosome (Chr) 15 and the similarity of the phenotype to the published *Cntn1* knockout phenotype, we analyzed *Cntn1* function in affected (presumed homozygous) mice. We assayed for CNTN1 protein by western blotting of brain and spinal cord tissue homogenates using standard techniques. Tissues were frozen in liquid nitrogen and stored at −80°C. The tissues were then homogenized in 1% NP-40 in phosphate buffered saline (PBS) supplemented with Protease Inhibitor Cocktail Tablets (Roche, Basal, Switzerland) using a PowerGen Model 125 Homogenizer (Fisher Scientific, Pittsburgh, PA) then centrifuged at 14,000 g for 10 minutes at 4°C. Cleared homogenates were then sonicated at 4°C and centrifuged again at 14,000 g for 10 minutes. Protein concentrations were assessed using a Bradford assay (BioRad, Hurcules, CA). 20 µg of protein was then analyzed by immunoblot. Protein lysates were resolved on Novex 10% Tris-Glycine Gels (Invitrogen, Carlsbad, CA) and transferred to an Invitrolon PVDF membrane for western blot analysis. Membranes were blocked with 5% skim milk in TBST (1× Tris-buffered saline, 0.1% Tween-20), and incubated overnight with CNTN1 goat polyclonal antibody (1∶200, R&D Systems, AF904) and β actin mouse monoclonal antibody clone AC-74 (1∶10,000, Sigma Aldrich, St. Louis, MO) diluted in blocking solution at 4°C. Following three 10 minute washes in TBST, the blots were incubated with the appropriate horseradish peroxidase-conjugated secondary antibodies (Jackson ImmunoResearch, West Grove, PA) diluted in blocking solution. After three 10-minute washes in TBST, the blots were developed using Western Lightning Plus-ECL, Enhanced Chemiluminescence Substrate (Perkin Elmer, Waltham, MA). We assayed for *Cntn1* transcription by amplifying the *Cntn1* gene from reverse transcribed brain cDNA using overlapping primer combinations that spanned the open reading frame. We also sequenced 11 of the 24 *Cntn1* exons in genomic DNA isolated from affected mice using standard Sanger sequencing methods. Because we were sequencing an already known and sequenced gene to look for causative mutations, no new sequence was generated. Therefore, we did not submit the sequence generated to GenBank.

### *In-vitro* contractile properties

Mice were anesthetized with isoflurane and the extensor digitorum longus (EDL) muscle was surgically exposed completely from distal to proximal tendon. Silk suture thread (5.0, Sofsilk, USA) was tied and secured with glue to form a small loop on each tendon. Once suture loops were in place, the muscle was completely freed and carefully removed and the anesthetized mouse was killed by cervical dislocation. Excised muscles were placed immediately in a custom organ bath filled with Krebs Ringer solution (NaCl 137 mM; NaHCO3 24 mM; D-glucose 11 mM; KCl 5 mM; CaCl2 2 mM; NaH2PO4H2O 1 mM; MgSO4 1 mM). The solution was bubbled with 5% CO2 in 95% oxygen, maintained at 25°C (Warner Instruments Model TC 324B, USA), and circulated continuously through the bath.

The sutures on proximal and distal tendons were attached, respectively, to a hook connected to a force transducer (Aurora Instruments, Dual Mode Lever System, Toronto, CA) and a post fixed permanently in the bath. Two platinum plate electrodes that flanked the muscle longitudinally were connected to a stimulator for delivery of supramaximal square-wave pulses (0.2 ms duration, Aurora Instruments, Biphase Current Stimulator, Toronto, CA) to produce isometric contractions. The force transducer is mounted on a micromanipulator that allows optimum muscle length (Lo) to be established by maximizing twitch force amplitude.

Once Lo was established, experiments consisted of eliciting and recording maximum isometric twitch force (Pt) from which measures of time to peak twitch (TPT) and half relaxation time (1/2 RT) were also derived. Twitches were elicited with single stimuli (0.2 ms duration, 0.5 pps). Isometric tetanic force (Po) was also recorded in response to a 4 pulse stimulus train at 80 pulses per second (PPS). Force data were digitized (Aurora Instruments, Toronto, CA) and recorded on a PC-based computer. Calipers were used to measure muscle length at Lo through a dissecting scope (Leica MZ6) mounted over the preparation. At the end of the experiment the muscle was removed from the bath and weighed.

## Results

### Phenotype

By 4–7 days after birth mutant mice are smaller than their littermates; by two weeks they appear emaciated and ataxic. About 80% of homozygous pups die by 18–20 days of age; the remaining 20% die by 30 days of age. Gross autopsies of two- and three-week-old pups revealed hardly any food in the stomach, intestines full of gas bubbles and pale livers. Frozen liver sections stained with oil red O showed fat deposits characteristic of fat mobilization in starving mice. Histopathologic screening of all tissues, as is routine in the MMR, failed to reveal obvious abnormalities. Likewise, no morphological anomalies were observed in brains stained with LFB and PAS. Calbindin immunostaining of cerebellar Purkinje cells showed normal dendritic arborization (data not shown) similar to knockout mice.

### Genetic Analysis

#### Mode of Inheritance

This mutation is inherited as an autosomal recessive. Heterozygotes are not visibly affected. The male from the original trio carrying the mutation was outcrossed to a B6C3H F1 (C57BL/6J×C3H/HeSnJ) female and produced one pair of normal appearing (+/?) F1 mice that subsequently produced 5 mutant mice and 30 normal mice. F1 mice from an outcross to C57BL/6J produced 60 mutant and 130 +/? mice and initial matings between heterozygotes in the colony (confirmed by the production of at least one affected mutant) produced 31 mutant and 90 +/? mice for a frequency of 27.7% (96 mutants/346 total mice), which is not statistically different from the expected 25%.

#### Chromosomal assignment

**Figure 1:**
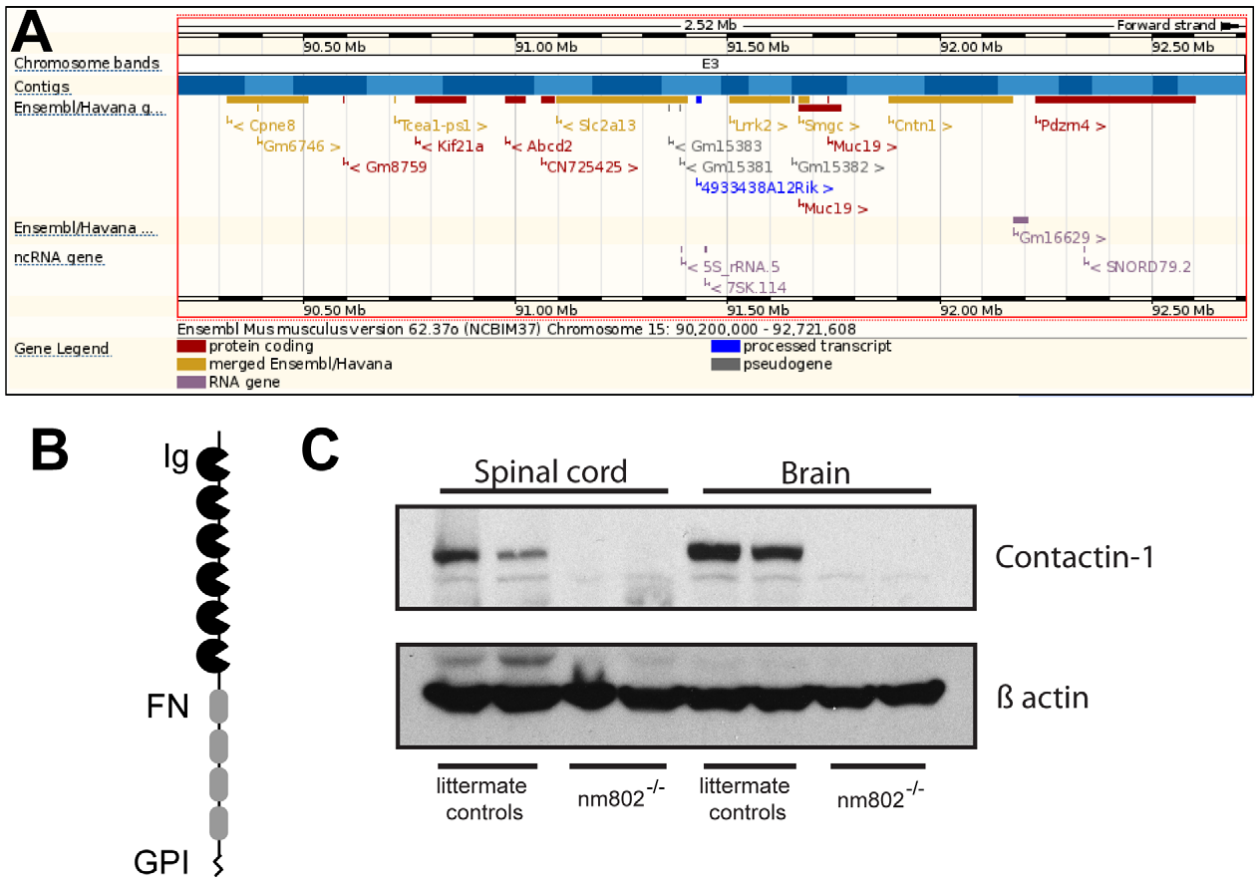
Contactin1 as a candidate gene for this mutation. A) The genetic interval of Chromosome 15 between *D15Mit242* and *D15Mit192* (2.5 Mb) is shown. Genomic features including protein-coding genes are indicated. The image is from the Ensembl genome browser (http://ensembl.org/index.html). B) The structure of CNTN1 is schematized; the protein has an N-terminal signal peptide for secretion, 6 immunoglobulin domains (Ig), 4 fibronectin repeats (FN), and a GPI-linked carboxy-terminal modification for attachment to membranes. C) Immunoblot of CNTN1 in spinal cord and brain from two unaffected littermate control mice and two affected mice reveals an absence of CNTN1 protein in the affected mice. β-actin was used as a loading control.

Initial linkage crosses in 1989 with visible markers revealed linkage with caracul (*Krt71^Ca^*) on Chr 15 (10.8±3.4; n = 303) in a backcross-intercross. A subsequent repulsion intercross with two other Chr 15 markers, underwhite (*Slc45a2^uw^*) and belted (*Adamts20^bt^*), confirmed the distal location on Chr 15 (*Slc45a2^uw^*–38.20±3.49 – new mutation, *Adamts20^bt^*; n = 584). In 2009, in an effort by MMR scientists to identify the mutated genes in old spontaneous mutations, this mutation was more precisely mapped using an intercross with DBA/2J. In this intercross the mutation was localized between *D15Mit242* at 90.2 Mb and *D15Mit192* at 92.7 Mb [*D15Mit242* – 1/104 chromosomes = .9% - new mutation – 3/104 = 2.9% - *D15Mit192*]. One of the eleven protein coding genes in this interval was the contactin 1 (*Cntn1*) gene spanning 91.8–92.1 Mb (Figure 1A). This region is conserved in human Chr 12 and human *CNTN1* maps at 12q11–q12 [22]. Two polymorphic SNPs flanking *Cntn1* (rs32088727 448 bp upstream and rs31768105 1889 bp downstream) were genotyped in the DNAs of the 4 mutant mice with recombinations between the MIT markers flanking the mutation. All 4 homozygous mutants were homozygous for the BALB parental *Cntn1* alleles.

### Candidate gene analysis

Given the similarity of the mutant phenotype to that of *Cntn1* knockout mice, and the lack of compelling evidence suggesting a mutation in one of the other genes in the interval might be causing the phenotype, we analyzed *Cntn1* in affected animals. CNTN1 is an Ig-superfamily cell adhesion molecule of 1020 amino acids that is known to cause neurodevelopmental phenotypes when mutated in mice [17] (Figure 1B). In cDNA prepared from mutant brain mRNA, it was very difficult to reliably amplify regions of the *Cntn1* open reading frame for sequencing, although other genes could be amplified from the same samples, suggesting a severe, specific decrease in *Cntn1* mRNA levels. Consistent with this, immunoblotting with an anti-CNTN1 antibody revealed a complete failure to detect CNTN1 protein in homogenates from affected mice, whereas a band of the appropriate size was readily detectable in samples from unaffected littermates (Figure 1C). The reduction of protein to levels below detection by western blotting indicates that the mutation in the *Cntn1* gene, which we have designated *Cntn1^J^*, is a null or very severe hypomorph. Although we have not identified the precise molecular lesion in the genomic DNA yet, with 11 exons from the 24 exon transcription unit sequenced with good coverage, we have strong evidence that the mutation is in *Cntn1*: 1) genome wide mapping localizes the mutation to an interval containing *Cntn1* on Chr. 15, 2) *Cntn1* and the mutant phenotype co-segregated in four animals with recombinations within the interval, 3) the phenotype closely resembles the reported *Cntn1* phenotype, and 4) *Cntn1* mRNA and protein are drastically reduced or absent. Thus, we feel it is very unlikely that the mutation could be in another gene in the interval with the secondary effect of eliminating *Cntn1* expression.

### Neuromuscular function assessment

**Figure 2:**
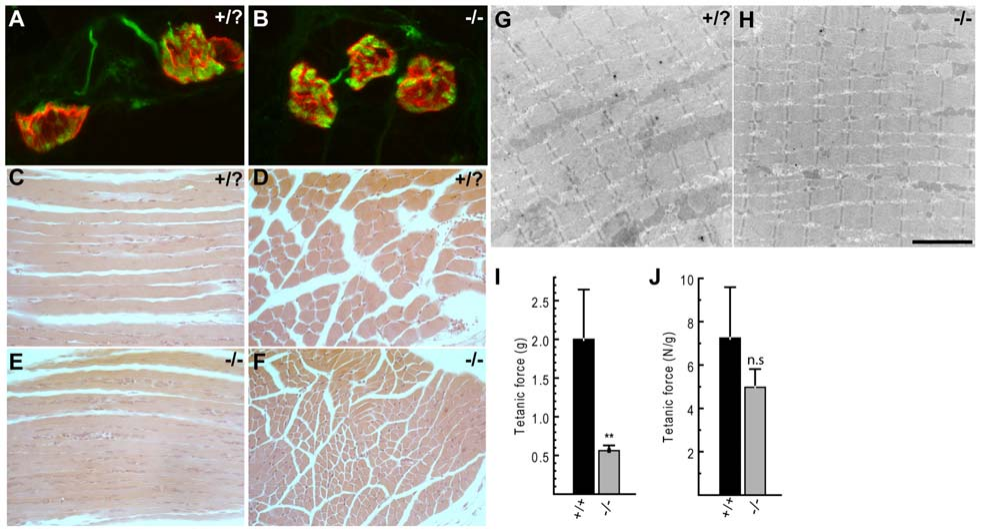
Neuromuscular analysis of *Cntn1* mutant mice. A) Neuromuscular junctions in wild type P11 mice have a plaque-like field of postsynaptic acetylcholine receptors (red) that is beginning to become convoluted. This is completely overlapped by the presynaptic motor nerve terminal (green). B) A similar NMJ morphology is seen in *Cntn1* mutant mice. Histological examination of longitudinal and cross sections of control (C,D), and mutant (E,F) hind limb muscles at P13 did not reveal hallmarks of myopathy. G,H) Transmission electron microscopy was used to evaluate sarcomere anatomy in the tibialis anterior muscle. The structure was not adversely affected by the mutation. I) The absolute maximal contractile force of the extensor digitorum longus muscle was reduced in mutant mice. J) When normalized for muscle weight, contractile force of mutant muscle was not significantly different than control. The scale bar in H represents 14 µm in A, B, 72 µm in C–F, and 3 µm in G, H.

Consistent with previous studies, neuromuscular junctions in *Cntn1* mutant animals had normal morphology [15] (Figure 2A, B). In control mice at postnatal day 11 (P11), the postsynaptic acetylcholine receptor field on the muscle membrane (labeled in red with α-bungartoxin) is becoming convoluted but has not yet assumed its mature pretzel-like shape. The motor nerve terminal (labeled in green with antibodies against neurofilament and SV2) fully overlaps the receptor field and fills the developing troughs in the muscle. A very similar morphology was observed in the mutant muscle, and there was no evidence of delayed maturation, partial or complete denervation, or dystrophic axons or nerve terminals. Similarly, histopathological examination of muscle did not reveal myopathy, fibrosis, neurogenic atrophy, or central myonuclei indicative of regenerating muscle fibers, although muscle fibers were smaller consistent with the smaller size of the mice (Figure 2 C–F). Similar results were obtained upon closer examination of muscle anatomy using transmission electron microscopy (Figure 2 G, H). Sarcomere structure and organization appeared normal in two mutant tibialis anterior muscles examined at P13. For example, sarcomeres were of uniform size and Z-lines were in register.

Such a lack of clear histopathological findings in mice with such a striking and severe phenotype typically indicates a functional deficit that does not impact anatomy. Since the human disease associated with *CNTN1* mutations is categorized as a congenital myopathy, we examined this functionally in muscle. Using isolated extensor digitorum longus (EDL) muscles from P13 mutant and control mice (N = 4 each), we tested muscle contractile properties by direct stimulation of the muscle. The mutant mice are dramatically smaller than littermates, and muscle weights were similarly smaller (∼60%) (Table 1). However, there was no reduction in the muscle weight∶body weight ratio, indicating that muscle is not disproportionately affected. Consistent with the reduced muscle weight, both the absolute twitch and tetanic force generated by the mutant muscles were significantly reduced compared to control (∼70%), but when normalized for muscle weight the force values were not different than control (Figure 2I, J and Table 1). The time to peak tension of the mutant muscles was also significantly slower (Table 1) and the half-relaxation time showed a slowing trend that was not significant. These changes may reflect secondary developmental differences in muscle proteins (e.g. myosin ATPase). Taken together, the histopathology, anatomy, and functional analysis of muscles in *Cntn1* mutant mice are inconsistent with a congenital myopathy, and suggest that the severe locomotor phenotype is the result of dysfunction in the nervous system, as previously suggested [17], [18], and not in the muscle itself.

**Table 1:** Additional *in vitro* contractile properties of directly stimulated EDL muscles.

	+/+	−/−
**Body weight (g) [BW]**	8.1±.0.2	3.3±0.4**
**Muscle weight (mg) [MW]**	2.7±0.2	1.1±0.2**
**MW:BW ratio %**	0.034±0.0023	0.033±0.0029
**Pt (g)**	1.2±0.4	0.32±0.06*
**Pt (N/g)**	4.3±1.5	2.9±0.9
**TPT (ms)**	21.1±1.1	25.1±2.6*
**1/2RT (ms)**	32.2±3.7	36.9±7.5

*<0.03.

**<0.0001.

**Figure 3:**
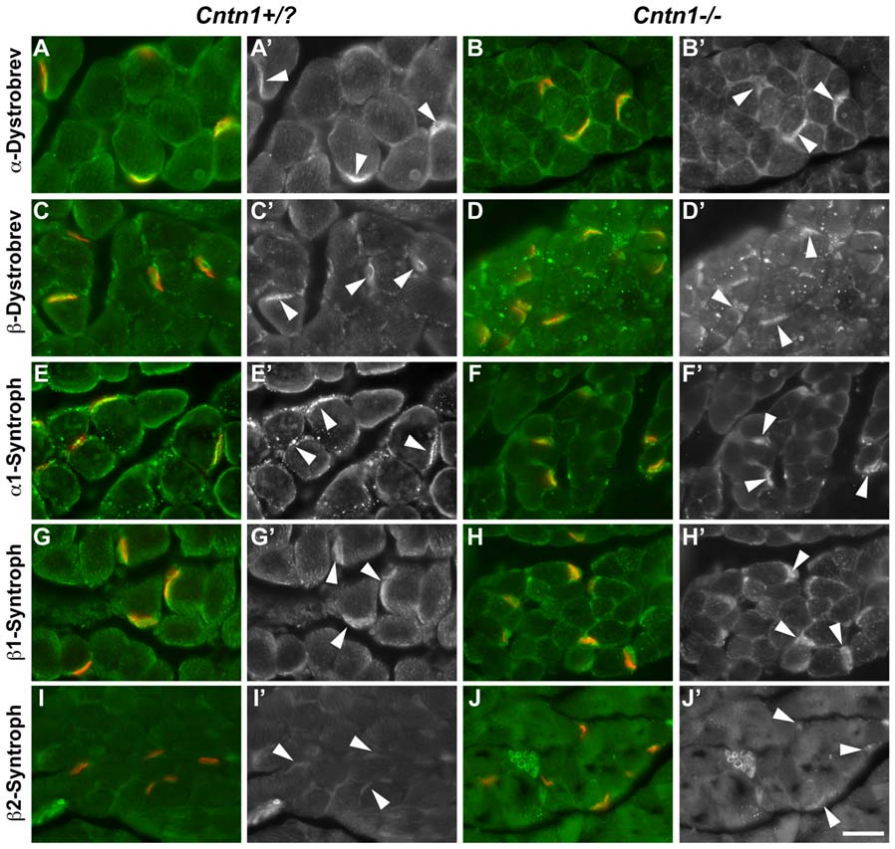
Dystrobrevin and Syntrophin localization in *Cntn1* mutant muscle. Cross sections of triceps surae from three mutant and three littermate control mice (ages P14, P15, and P16) were labeled with antibodies recognizing dystrobrevins and syntrophins (green). NMJs were counterstained with α-bungarotoxin (red). Merged images as well as the green channel with NMJs denoted by arrowheads are shown. Immunolabels are noted at left, genotypes are noted at top (control left, mutant right). The scale bar in the lower right is 25 µm.

Muscle twitches recorded in mutant mice are smaller and have a longer time-to-peak tension. Reduced absolute values are due to smaller muscles in mutants because normalized twitch forces are not different from wild-type. Similar outcome is shown for tetanic force in Figure 3. MW:BW ratio%, percent muscle to body-weight; Pt(g), absolute twitch force; Pt (N/g), twitch force normalized to muscle weight; TPT, time-to-peak twitch tension; 1/2RT-twitch half-relaxation time; N, Newtons (force); ms, milliseconds.

Another feature of the myopathic patients is an alteration in dystrobrevins and syntrophins, intracellular proteins associated with the dystroglycan-glycoprotein complex in muscle [15], [16]. Specifically, α1- and β1-syntrophin show their normal localization to the muscle membrane; however, β2-syntrophin and α-dystrobrevin are absent from the muscle membrane of patients, although α-dystrobrevin is retained at the NMJ. Such changes were not observed in the previously reported mice lacking *Cntn1* [15]. To determine if the spontaneous allele of *Cntn1* in a BALB/c genetic background had altered dystrobrevin and syntrophin localization in muscle, we stained muscle cross sections with antibodies to α- and β-dystrobrevin and α1-, β1-, and β2-syntrophin (Figure 3). Alpha-dystrobrevin was still clearly localized at the muscle membrane and enriched at NMJs (labeled with α-bungarotoxin) in both control and mutant samples (Figure 3 A, B). Beta-dystrobrevin, α1-syntrophin and β1-syntrophin all showed a similar distribution and NMJ enrichment that did not differ markedly between control and mutant samples (Figure 3 C–H). Staining for β2-syntrophin was weak even in control samples, but staining at the NMJ was detectable in mutant muscles, although extrasynaptic labeling was below detection (Figure 3 I, J).

### *Cntn1* expression in the retina

**Figure 4:**
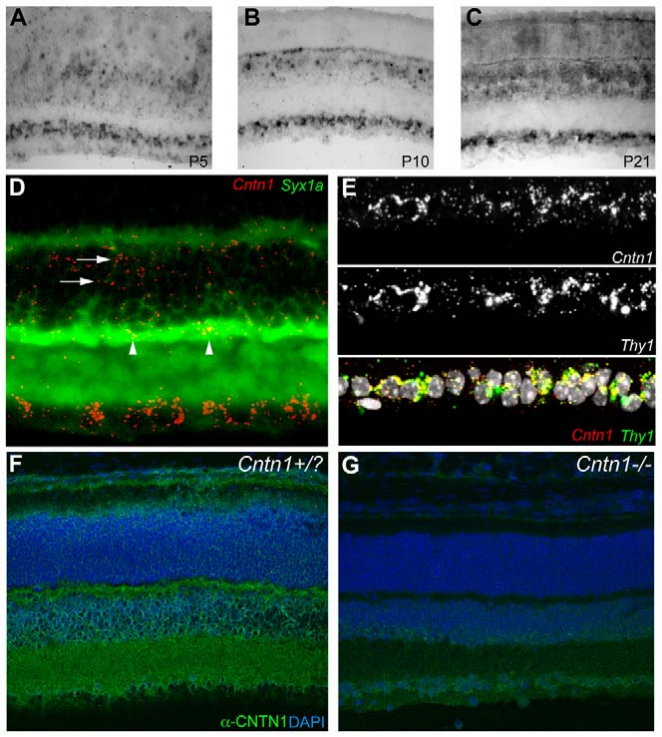
Expression and localization of *Cntn1* in the retina. A–C *In situ* hybridization for *Cntn1* expression in the wild type mouse retina at P5 (A), P10 (B), and P21 (C). A subset of cells in the retinal ganglion cell layer (bottom) and inner nuclear layer are positive for *Cntn1* expression. Photoreceptors in the outer nuclear layer (top) do not have signals above background. D) Double label *in situ* hybridization with *Cntn1* and syntaxin1a at P21 demonstrates that some amacrine cells in the inner nuclear layer are positive for *Cntn1* expression (arrowheads). Other *Cntn1*-positive cells are likely to be bipolar cells based on their position (arrows). E) In the retinal ganglion cell layer, a majority of cells expressing *Cntn1* at P21 also express *Thy1*, a marker of ganglion cells. F) Immunolabeling of retinas with anti-CNTN1 antibodies at P14 revealed strong labeling of the synaptic plexiform layers, as well as immunoreactivity in the cellular layers, particularly the inner nuclear layer. G) Immunolabeling retinas from *Cntn1* mutant mice revealed a marked reduction, but not an elimination of signal intensity in images collected with equivalent parameters.

The retina provides an excellent system for examining neurodevelopmental phenotypes because of its highly reproducible anatomy and the availability of markers for numerous specific neuronal cell types. Furthermore, a possible function for *Cntn1* in the retina has not been previously reported. We therefore examined the retinas of the *Cntn1* mutant mice; however, we first determined the expression pattern of *Cntn1* in wild type retinas to better inform the choice of markers to be used to look for phenotypes in the *Cntn1* mutant retinas. By *in situ* hybridization, *Cntn1* is expressed by many but not all cells in the retinal ganglion cell (RGC) layer (ganglion cells and amacrine cells) and in the inner nuclear layer (amacrine, bipolar, horizontal cells, and Muller glial cells). No expression above background was seen in the outer nuclear layer (photoreceptors). This pattern was observed in the developing retina (P5), and persisted through eye opening and in the mature retina (P21, figure 4 A–C). Double *in situ* hybridization with *Cntn1* and syntaxin 1A (*Syx1a*), a marker of amacrine cells, demonstrated that some amacrine cells do express *Cntn1*, but additional cells in the inner nuclear layer (probably bipolar cells based on their position) also express *Cntn1* (Figure 4D). In the retinal ganglion cell layer, double *in situ* hybridization with probes against *Thy1*, a marker of RGCs, showed that most *Cntn1*-positive cells in the ganglion cell layer are indeed ganglion cells (as opposed to misplaced amacrine cells, Figure 4E).

Immunofluorescent labeling of CNTN1 protein in two-week-old retinas revealed strong staining in the inner and outer plexiform layers of the retina, as well as in the inner nuclear layer, consistent with CNTN1 localization on the processes of ganglion, amacrine, and bipolar neurons (Figure 4F). The anti-CNTN1 labeling was somewhat dependent on fixation and staining conditions (see methods), although the conclusion that CNTN1 is abundantly expressed in the retina and largely localized to the plexiform layers was consistent. The specificity of the immunolabeling was tested by staining sections from mutant retinas prepared, labeled, and imaged in parallel with littermate control samples. The signal was markedly reduced in the retinas of mutant mice, but not eliminated (Figure 4G). Given the absence of signal in western blots from the mutant animals, this residual staining may reflect nonspecific antibody background, or a low level of CNTN1 protein still being produced by this allele. Considering the sensitivity and excellent signal to noise ratio of the western blot, we prefer the former explanation, but we cannot rule out the latter. Regardless, these results indicate that numerous cell types in the mouse retina express *Cntn1*, raising the possibility of a developmental phenotype in the retina in the *Cntn1* mutant animals.

### Normal retinal development in *Cntn1* mutant mice

**Figure 5:**
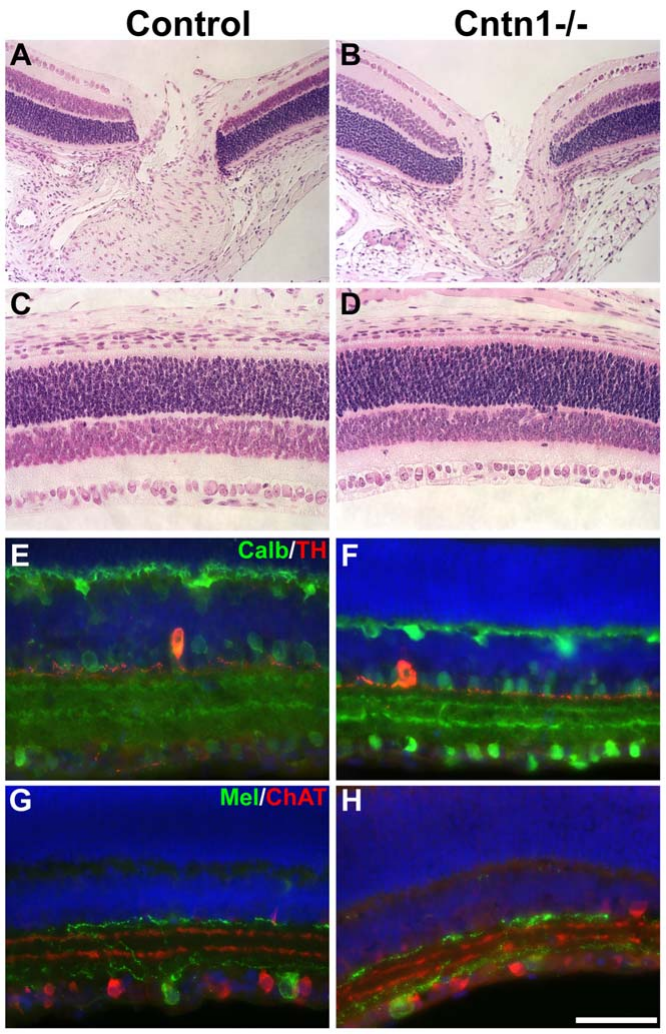
Normal retina development in *Cntn1* mutant mice. A,B) The retina and optic nerve head of wild type and *Cntn1* mutant mice stained with H&E did not reveal any defects. C,D) More peripheral areas of the retina were also normal in the mutant. E,F) Staining for dopaminergic amacrine cells (red, anti-tyrosine hydroxylase) and horizontal cells and calbindin-positive amacrine cells (anti-calbindin, green), showed that cell bodies and dendritic arbors of these cells were in the appropriate anatomical location. G,H) Staining for intrinsically photoresponsive ganglion cells (anti-melanopsin, green) and starburst amacrine cells (anti-choline acetyltransferase, red) showed that these cells were also unaffected by the loss of *Cntn1*. The scale bar in H represents 145 µm in A–D and 72 µm in E–H.

The broad expression of *Cntn1* in the retina suggests a functional role in retinal development. In addition, other Ig-superfamily members with very similar extracellular domain structures, such as the DSCAMs or Sidekicks, cause developmental defects in the mouse and chick retina when mutated [23]–[26]. Since it is unknown if the full scope of *Cntn1* neurodevelopmental phenotypes has been described, we examined retinas in *Cntn1* mutant mice to determine if similar defects would be evident. Upon histological examination, the normal layering of the retina was present and was well organized. Defects were not seen in the peripheral retina or at the optic nerve head, the point at which retinal ganglion cell axons exit the eye to form the optic nerve (Figure 5 A–D). Antibodies recognizing specific cell types in the retina were also used to label sections. This is a more sensitive examination of retinal anatomy that can detect misplaced cell bodies that may have failed to migrate properly, supernumerary or under-represented cells of a given type, and defects in dendrite arborization, which normally is restricted to specific strata in the synaptic plexiform layers for a given cell type. No defects in retinal anatomy were detected. The following cell types were examined: AII amacrine cells (Disabled1-positive), intrinsically photoresponsive retinal ganglion cells (melanopsin-positive), horizontal cells (calbindin positive), starburst amacrine cells (choline acetyltransferase-positive), bNOS-positive amacrine cells, rod bipolar cells (protein kinase C-alpha-positive), alpha retinal ganglion cells (SMI-32/neurofilament-positive), and dopaminergic amacrine cells (tyrosine hydroxylase-positive). Similar results were obtained in an independent examination of *Cntn1* mutant retinas, which additionally determined that glycinergic amacrine cells (VGLUT3-positive) and a mixed population of monostratified retinal ganglion cells (KV4.2-positive) were also normal in the absence of *Cntn1*. Representative images of intrinsically photoresponsive retinal ganglion cells, starburst amacrine cells, and dopaminergic amacrine cells are shown (Figure 5 E–H). Thus, the loss of *Cntn1* does not impact the retina as prominently as other related proteins such as DSCAMs, but subtle defects in other cell types may have escaped detection.

## Discussion

We have identified a new, spontaneous mutation in BALB/c mice that maps to Chromosome 15 and results in a null allele of *Cntn1*. The phenotype of these mice closely resembles the reported phenotype of *Cntn1* targeted knockout mice [17]. The homozygous animals are smaller than littermates by a week of age, have abnormal locomotor function, and die by a few weeks of age. Neuromuscular junctions and muscle in the homozygous animals are anatomically normal, and an examination of the retina for other neurodevelopmental phenotypes did not reveal any defects. These results indicate that the loss of *Cntn1* causes a severe and early onset phenotype that is consistent across multiple genetic backgrounds, but that the phenotype in mice differs from human Compton-North congenital myopathy in the backgrounds studied to date.

The difference in phenotype in mice and humans presents several interesting questions. Although we do not favor this hypothesis, it is a possibility that the *CNTN1* mutation identified in patients is not the cause of the lethal congenital myopathy. The mutation was found in three affected individuals in a single consanguineous family [15]. Mapping localized the mutation to an interval on Chromosome 12 containing *CNTN1* and 75 other protein-coding genes. Expression analysis from muscle indicated 14 genes in this interval were altered in their expression in affected individuals, but *CNTN1* showed the greatest change, with undetectable expression in the affected patients. Of the fourteen genes, seven were sequenced (as well as 18 other candidates) and no changes were found except in *CNTN1*. In addition to the undetectable level of expression, *CNTN1* contained an early frame shift mutation, making it the strongest candidate gene. For the *CNTN1* mutation to not be the causative factor, one would have to invoke a second closely linked mutation that results in congenital lethal myopathy before a later *CNTN1* phenotype could arise. This seems very unlikely. Another interesting possibility is that a truncated amino-terminal fragment of CNTN1 may be produced in these patients, and may cause a pathological effect on muscle. As more alleles of *CNTN1* are identified in humans, it will be interesting to see if the severe myopathy is a reproducible feature of the phenotype, or if it is selectively found in truncating mutations like S291fsX296. The mouse muscle does show a trend toward reduced force, even when normalized for its smaller size. Our stimulation protocol was chosen because it approximates physiological conditions, other more demanding protocols may have revealed decreased contractile function in the mutant muscle that may be indicative of a myopathy. Finally, it may be misleading to think of the mouse myopathy phenotype as less severe or absent, when the situation may be that the mouse neurological phenotype is more severe and proves lethal before the myopathy phenotype is evident.

Perhaps the most likely explanation for the discrepancy between the mouse and human phenotypes is simply species differences. This may reflect a difference in expression pattern. The mouse gene is not abundantly expressed in muscle, whereas CNTN1 was detectable on microarrays from control patient muscle biopsies. Similarly, mice may be better able to compensate for the loss of *Cntn1* by upregulating related genes, such as *Cntn2-4*. Finally, the resistance of mouse muscles to degeneration is seen in other mutations, such as dystrophin, mutations, which cause severe Duchenne muscular dystrophy in people, but a comparatively mild phenotype in the *mdx/mdx* mouse. These differences in severity may be a function of the smaller size or decreased physical loads in mouse muscles, or may represent a more fundamental difference in muscle biology between mice and humans.

While it remains to be determined if mouse *Cntn1* mutations are a good model of human *CNTN1*-associated disease, the striking phenotype of the *Cntn1* mutant mice indicates that they are still an excellent system for understanding the biology of CNTN1 function at the cellular and molecular levels. For example, mouse CNTN1 is critically involved in the formation of septate-like junctions and the localization of ion channels to the node and paranodal regions of peripheral Schwann cells [18], [27]. The molecular interactions underlying these functions will undoubtedly shed light on the function of CNTN1 in general in other regions of the central nervous system or in other tissues. Consistent with this, *Cntn1* mutant mice have defects in the hypothalamus that may impact feeding behavior, but hand feeding did little to prolong lifespan indicating that this is only part of the neurological impairment affecting these mice [28].

The new mutation in *Cntn1* identified in this study further confirms the important role for this gene in mouse development and indicates that the phenotype resulting from the loss of *Cntn1* is not highly sensitive to genetic background effects. Furthermore, these studies indicate that other neurodevelopmental processes, such as stratification of the retina, are not affected by the loss of *Cntn1*. Discrepancies between the mouse and human phenotype merit further investigation. If the primary phenotype of *CNTN1* mutations in people is a myopathy, then understanding why this is not a prominent aspect of the mouse phenotype may lead to possible therapeutic strategies for *CNTN1*-related diseases.
